# Untargeted metabolomics reveals metabolic reprogramming and key metabolites in adherent and suspension Vero cells

**DOI:** 10.3389/fcell.2026.1936095

**Published:** 2026-08-28

**Authors:** Wenna Ji, Ziyi Chen, Xinyu Yue, Zhengqing Ma, Zhenbin Liu, Di Yang, Na Sun, Rui Ma, Zilin Qiao, Jianguo Chen, Jiamin Wang

**Affiliations:** 1 Ministry of Education Engineering Research Center for Key Technologies and Industrialization of Cell-Based Vaccines, Northwest Minzu University, Lanzhou, China; 2 Gansu Animal Cell Technology Innovation Center, Northwest Minzu University, Lanzhou, China; 3 School of Bioengineering/Biomedical Research Center, Northwest Minzu University, Lanzhou, China; 4 State Ethnic Affairs Commission Key Laboratory of Bioengineering and Technology, Northwest Minzu University, Lanzhou, China

**Keywords:** differential metabolites (DAMs), metabolic reprogramming, metabolomics, suspension adaptation, Vero cell

## Abstract

**Introduction:**

Vero cells are the primary host for industrial vaccine production, and high-density suspension culture is the predominant manufacturing approach. However, this method faces major challenges, including low shear tolerance, difficulty adapting to serum-free conditions, metabolic imbalance, prolonged acclimation periods, and batch-to-batch variability. Furthermore, the metabolic reprogramming mechanisms underlying suspension adaptation remain poorly understood.

**Methods:**

We established serum-free suspension adaptation of adherent Vero cells using reduction serum levels strategy. Biological characteristics of suspension-adapted cells were analyzed through morphological observation, cell viability assays, and karyotyping. A recombinant rabies vesicular stomatitis virus infection model was used to assess viral susceptibility and replication capacity. Untargeted liquid chromatography–tandem mass spectrometry metabolomics was then utilized to systematically characterize intracellular metabolic profiles of Vero cells cultured under adherent and suspension conditions. Differential metabolites were identified, and key regulatory pathways were evaluated to elucidate the metabolic mechanisms of suspension adaptation and identify potential optimization targets.

**Results:**

Suspension-adapted Vero cells exhibited uniform morphology, stable genetic characteristics, and robust proliferative capacity, without evidence of degeneration or mutation. Viral sensitivity assays demonstrated efficient viral adsorption and replication, yielding stable viral titers and confirming suitability for vaccine production. Metabolomic analysis identified 119 metabolites (32 upregulated, 47 downregulated, and 40 unchanged), with significant enrichment in alanine/aspartate/glutamate metabolism, glycine/serine/threonine metabolism, and the tricarboxylic acid cycle. Suspension cells displayed Warburg-like metabolic features, including enhanced glycolysis, increased lactate accumulation, and pronounced upregulation of succinate and malate. L-serine and ethanolamine phosphate were strongly upregulated, whereas polyunsaturated fatty acids were significantly reduced. Suspension adaptation involved coordinated remodeling of energy, amino acid, nucleotide, and lipid metabolism; enhancing shear tolerance through membrane lipid remodeling; improving antioxidant capacity via serine metabolism; and supporting high-density proliferation through glycolytic reprogramming.

**Discussion:**

This study established a serum-free suspension adaptation system for Vero cells with stable phenotypic characteristics and high viral susceptibility while elucidating metabolic regulatory mechanisms that underlie suspension adaptation. The findings provide metabolic targets and a theoretical foundation for rational medium design, targeted supplementation strategies, and development of genetically engineered cell lines with enhanced shear resistance.

## Introduction

Vero cells (Verda Reno cells; African green monkey kidney cells), a classic continuous cell line certified by the World Health Organization, have served as the primary host cells for viral vaccine research, development, and production for decades. They are widely used in the manufacture of vaccines against poliovirus (Vidor et al., 1997; Okemoto-Nakamura et al., 2021), influenza virus (Chan and Tambyah, 2012; Smith et al., 2013), rabies virus (Yokomizo et al., 2004; Li et al., 2024), and Zika virus (Wressnigg et al., 2024); they are also valuable in basic research fields such as cell biology, virology, and toxicology (Kiesslich and Kamen, 2020). Conventional Vero cells exhibit adherent growth and depend on culture substrates for proliferation. Because available surface area limits cell expansion, adherent cultures often struggle to meet the demands of large-scale industrial vaccine production. In contrast, suspension culture eliminates the need for cell-adherent substrates and enables high-density, large-scale expansion in stirred bioreactors, thus improving production efficiency and reducing costs. Consequently, suspension culture represents a key direction for future industrial vaccine manufacturing (Zhang et al., 2023). However, mechanisms underlying the adaptation of Vero cells from adherent to suspension culture are not fully understood. To our knowledge, systematic analyses of differences in cellular physiology, energy and material metabolism, and key regulatory pathways between these culture modes have not been published, limiting the precise optimization of suspension culture systems.

As an integral branch of systems biology, metabolomics enables comprehensive qualitative and quantitative analysis of intracellular small-molecule metabolites, providing dynamic insights into cellular metabolism, energy supply, and stress regulation under various culture conditions (Zhang et al., 2013). It is widely used to monitor cell culture processes, investigate mechanisms underlying phenotypic variation, and optimize culture systems. Early studies of animal cell metabolism primarily focused on monitoring core nutrients such as glucose and glutamine, as well as metabolic byproducts including lactate and ammonia. The goal was to characterize nutrient utilization patterns, identify optimal media formulations and supplements, and support rational medium design. For example, Chong et al. (2009) conducted dynamic metabolomic analysis of Chinese hamster ovary (CHO) cells during fed-batch culture; they found that harmful amino acid derivatives, including N-acetyl-L-phenylalanine and N,N-dimethyl-formamidine, accumulated over time, whereas essential nutrients such as tryptophan and choline were rapidly depleted. These findings demonstrated the value of metabolomics for guiding medium optimization to improve cell growth and antibody production. Similarly, (Sun et al., 2023) combined untargeted and targeted metabolomics to identify significant differences in choline, tryptophan, purine/pyrimidine metabolism, and the pentose phosphate pathway. Suspension-cultured cells exhibited reduced proliferation and specific growth rates, accompanied by decreased levels of choline, tryptophan, and tyrosine. Exogenous supplementation with choline chloride partially restored suspension cell growth. With the continued advancement of metabolomics technologies, including liquid chromatography–tandem mass spectrometry (LC-MS/MS), gas chromatography–mass spectrometry, and nuclear magnetic resonance, research has shifted from process optimization toward elucidating intracellular metabolic regulation. These techniques enable detailed characterization of intracellular metabolic states and pathway alterations associated with key cellular phenotypes, such as growth, proliferation, and clonal stability, thereby providing powerful tools to understand mechanisms that underlie efficient cell expansion and protein expression (Torres et al., 2023). For example, (Jang et al., 2022) used targeted metabolomics to analyze human embryonic kidney (HEK293) cells and found that adherent cultures exhibited higher cell density, growth rate, and specific growth rate, along with elevated levels of central carbon metabolites involved in glycolysis, the tricarboxylic acid (TCA) cycle, the pentose phosphate pathway, amino acid metabolism, and nucleotide metabolism. In contrast, suspension cells showed robust accumulation of itaconic acid and succinic acid, accompanied by an increased adenosine monophosphate (AMP)/adenosine triphosphate (ATP) ratio, highlighting distinct metabolic adaptations between culture modes. However, systematic studies of the global metabolic profiles of Vero cells under adherent and suspension conditions, including identification of key differential metabolites and the regulatory mechanisms of core metabolic pathways, remain limited. Consequently, a metabolic framework capable of directly guiding suspension culture optimization has not yet been established. Here, we utilized a direct approach to achieve serum-free suspension adaptation of adherent Vero cells. The biological characteristics of suspension-adapted cells were evaluated through morphological observation, cell viability assays, and karyotype analysis. In parallel, a recombinant rabies vesicular stomatitis virus (VSV) infection model was used to assess viral susceptibility and replication capacity. We then systematically compared the metabolomic profiles of adherent and suspension cultures, identified key differential metabolites, and analyzed enriched metabolic pathways to elucidate regulatory mechanisms that underlie adaptation to various culture modes. The findings provide a theoretical foundation and technical support for precise optimization of Vero cell suspension culture, high-density cultivation, and improved vaccine production efficiency.

## Methods

### Materials

Adherent Vero cells (Vero-AD), preserved by the Gansu Provincial Animal Cell Technology Innovation Center, were passaged in M199 medium (Lanzhou Bailin Biotechnology Co., Ltd.). Suspension Vero cells (Vero-XF) were maintained in an equal-volume mixture of Vero602 (Zhejiang YSK Biotechnology Co., Ltd.) and Vero-S (Shanghai Basalmedia Co., Ltd.). Recombinant VSV expressing the rabies virus glycoprotein (rVSV-RABV-G) was provided by the Engineering Research Center of the Ministry of Education for Key Technologies and Industrialization of Cell-Based Vaccines.

### Cell culture and suspension adaptation

Adherent cell culture: Vero adherent cells were cultured at 37 °C with 5% CO_2_ until a confluent monolayer formed. The medium was removed, and cells were washed 2–3 times with phosphate-buffered saline. After removal of phosphate-buffered saline, cells were digested with 0.25% trypsin at room temperature for 1–2 min. The flask was gently tapped to detach cells, and fresh medium was added to terminate digestion. Cells were resuspended by gentle pipetting and passaged at a 1:6 ratio. After 1–2 stable passages, cells were used for subsequent experiments.

Suspension adaptation: Adherent Vero cells in the logarithmic growth phase were digested with 0.25% trypsin, collected by centrifugation, resuspended in suspension medium, and adjusted to a density of 2 × 10^6^ cells/mL. Cells were cultured on a shaker at 100 rpm in a humidified incubator at 37 °C with 5% CO_2_. Cell morphology, density, and viability were monitored daily; adaptation was continued until stable suspension growth was achieved with viability ≥90%.

### Biological characteristic assays

Growth curves and cell metabolism analysis: Logarithmic-phase adherent Vero cells exhibiting ≥90% confluence digested with 0.25% trypsin, counted, and seeded in 24-well plates at 2 × 10^4^ cells/mL. Cells were cultured at 37 °C with 5% CO_2_. Beginning 24 h after seeding, three replicate wells were sampled daily until cell density substantially declined. Culture supernatants were collected and stored at 2 °C–8 °C for glucose and lactate analysis; residual cells were detached, stained with trypan blue, and assessed for density and viability. Concerning suspension Vero cells (viability ≥90%), cultures were adjusted to 2 × 10^6^ cells/mL and maintained in 125-mL shake flasks at 37 °C, 5% CO_2_, and 100 rpm. Samples were collected every 24 h, then centrifuged for supernatant collection and viability assessment. Growth and metabolic curves were generated based on culture time, cell density, viability, and glucose/lactate levels. Cell doubling time, maximum cell density, glucose consumption, and lactate production rates were calculated to compare the growth and metabolic characteristics of adherent and suspension Vero cells.

Soft agar colony formation assay: A two-layer soft agar system was prepared using sterile low-melting-point agarose. A 0.7% agar bottom layer was added to six-well plates and allowed to solidify at room temperature. Adherent Vero cells were detached to generate single-cell suspensions, whereas suspension Vero cells were collected directly. Cells were adjusted to 5 × 10^3^ cells/mL, mixed with prewarmed 1% top agarose medium, and overlaid onto the solidified bottom layer. After complete solidification, plates were incubated at 37 °C with 5% CO_2_ for 14–21 days. Fresh complete medium was added every 3–4 days to support cell growth.

Chromosome analysis: Adherent Vero cells were seeded in T75 flasks at a 1:6 passage ratio and cultured to approximately 90% confluence. Suspension Vero cells were collected after 24 h of culture with medium renewal. Both cell types were treated with colchicine for 4–6 h to arrest cells in metaphase. Adherent cells were maintained under static conditions, whereas suspension cells were cultured in a shaking incubator (100 rpm, 37 °C, 5% CO_2_). Cells were harvested by centrifugation and subjected to hypotonic treatment with prewarmed hypotonic solution at 37 °C for 30 min. Samples were then fixed 2–3 times using freshly prepared fixative; each fixation lasted at least 30 min. After slide preparation and air drying, cells were subjected to Giemsa staining. At least 50 metaphase cells were examined microscopically for chromosome counting and statistical analysis.

Virus sensitivity analysis: Adherent Vero cells in good condition were detached, seeded at 2 × 10^4^ cells/mL, and cultured to 80%–90% confluence prior to viral susceptibility testing. For the 50% tissue culture infectious dose (TCID_50_) assay, recombinant virus was serially diluted in M199 medium from 10^−4^ to 10^−12^, with a blank control included in parallel. Diluted virus preparations were inoculated onto Vero cell monolayers and incubated at 37 °C with 5% CO_2_. Cytopathic effects were monitored daily. Positive and negative wells were recorded; TCID_50_ values were calculated using standard methods.

### Metabolite extraction

Cell pellets were thawed at 4 °C, and 25-mg aliquots were transferred to 1.5-mL microcentrifuge tubes. Each sample was mixed with 800 μL precooled extraction solvent (methanol/acetonitrile/water, 2:2:1, v/v/v; −20 °C), 10 μL internal standard, and two steel beads. Samples were homogenized at 50 Hz for 5 min, sonicated in a 4 °C water bath for 10 min, and incubated at −20 °C for 1 h. After centrifugation at 25,000 rpm and 4 °C for 15 min, supernatant (600 μL) was collected and vacuum-dried. Residues were reconstituted in 200 μL methanol/water (1:9, v/v), vortexed for 1 min, and sonicated again at 4 °C for 10 min. Following a second identical centrifugation step, supernatants were transferred to LC vials. Quality control (QC) samples were prepared by pooling equal volumes (20 μL) from each sample to assess the repeatability and stability of LC-MS/MS analysis.

### LC-MS/MS profiling

Metabolite separation and detection were performed using a Waters 2D UPLC system (Waters, United States of America) coupled to a Q Exactive HF high-resolution tandem mass spectrometer (Thermo Fisher Scientific, United States of America). A BEH C18 column (1.7 μm, 2.1 × 100 mm; Waters) was used for chromatographic separation. In positive ion mode, the mobile phase consisted of 0.1% formic acid in water (Solvent A) and 0.1% formic acid in methanol (Solvent B). In negative ion mode, the mobile phase consisted of 10 mM ammonium formate in water (Solvent A) and 10 mM ammonium formate in 95% methanol (Solvent B). The elution gradient was as follows: 0–1 min, 2% B; 1–9 min, 2%–98% B; 9–12 min, 98% B; 12–12.1 min, 98%–2% B; and 12.1–15 min, 2% B. The flow rate was 0.35 mL/min, column temperature was 45 °C, and injection volume was 5 μL.

Primary and secondary mass spectrometry data were acquired using the Q Exactive HF mass spectrometer. The scan range was m/z 70–1050, with a full-scan resolution of 120,000, automatic gain control target of 3 × 10^6^, and maximum injection time of 100 m. The three most intense precursor ions were selected for fragmentation and MS/MS acquisition. MS/MS resolution was set to 30,000, automatic gain control target to 1 × 10^5^, and maximum injection time to 50 m. Stepped collision energies of 20, 40, and 60 eV were applied. Electrospray ionization source parameters were as follows: sheath gas flow rate, 40; auxiliary gas flow rate, 10; spray voltage, 3.80 kV in positive ion mode and 3.20 kV in negative ion mode; capillary temperature, 320 °C; and auxiliary gas heater temperature, 350 °C. To minimize systematic bias, samples were analyzed in random order, and one QC sample was injected after every 10 study samples.

### Omics data analysis

Raw data preprocessing: Mass spectrometry data were processed using Compound Discoverer 3.1 (Thermo Fisher Scientific) for peak identification, alignment, and noise reduction. Data normalization, QC-based metabolite filtering, and statistical analyses were performed using the metaX R package (version 1.2.3) (Wen et al., 2017) in R version 4.2.1 to generate a high-quality quantitative dataset.

Data merging: To maximize coverage of endogenous metabolites with diverse physicochemical properties, mass spectrometry data were acquired in both positive-ion (pos) and negative-ion (neg) modes. Pos mode is particularly suitable for detecting nitrogen-containing basic compounds, amino acid derivatives, and nucleosides; neg mode provides sensitive detection of acidic metabolites such as organic acids, phosphates, fatty acids, and carbohydrates. To minimize mode-specific bias and improve metabolite coverage, metabolites identified in both modes were merged and deduplicated before differential metabolite screening, expression profiling, and Kyoto Encyclopedia of Genes and Genomes (KEGG) (Kanehisa et al., 2023) pathway enrichment analysis.

Multivariate statistical analysis: Principal component analysis (PCA) and partial least squares-discriminant analysis (PLS-DA) were performed to evaluate global metabolomic differences between adherent and suspension cultures, then identify potential differential metabolites.

Differential metabolite screening and analysis: Differential metabolite analysis was performed using Student’s t-test. The Benjamini–Hochberg false discovery rate (FDR) method was utilized to correct for multiple testing, and adjusted q values were calculated. Metabolites satisfying all three criteria were considered as differential metabolites: variable importance in projection (VIP) ≥ 1 (from the first two components of PLS-DA), fold-change ≥2 or ≤0.05, and FDR-corrected q < 0.05. For KEGG pathway enrichment analysis of differential metabolites, hypergeometric test was performed and pathways with raw *P* < 0.05 were regarded as significantly enriched. Cluster analysis was performed after log_2_ transformation and z-score normalization. Hierarchical clustering with Euclidean distance was used to evaluate metabolite expression patterns. KEGG-based pathway enrichment analysis was conducted to identify significantly altered metabolic pathways.

### Statistical analysis

Data were analyzed using SPSS version 26.0 (IBM, United States of America) and GraphPad Prism version 9.5 (GraphPad Software, United States of America). Quantitative data are presented as mean ± standard deviation. Intergroup comparisons were performed using t-tests or analysis of variance; *p*-values <0.05 were considered statistically significant. Pearson correlation analysis was conducted to evaluate relationships among metabolites, and the results were visualized using correlation heatmaps and network diagrams.

## Results

### Establishment of a suspension Vero cell culture system

**FIGURE 1:**
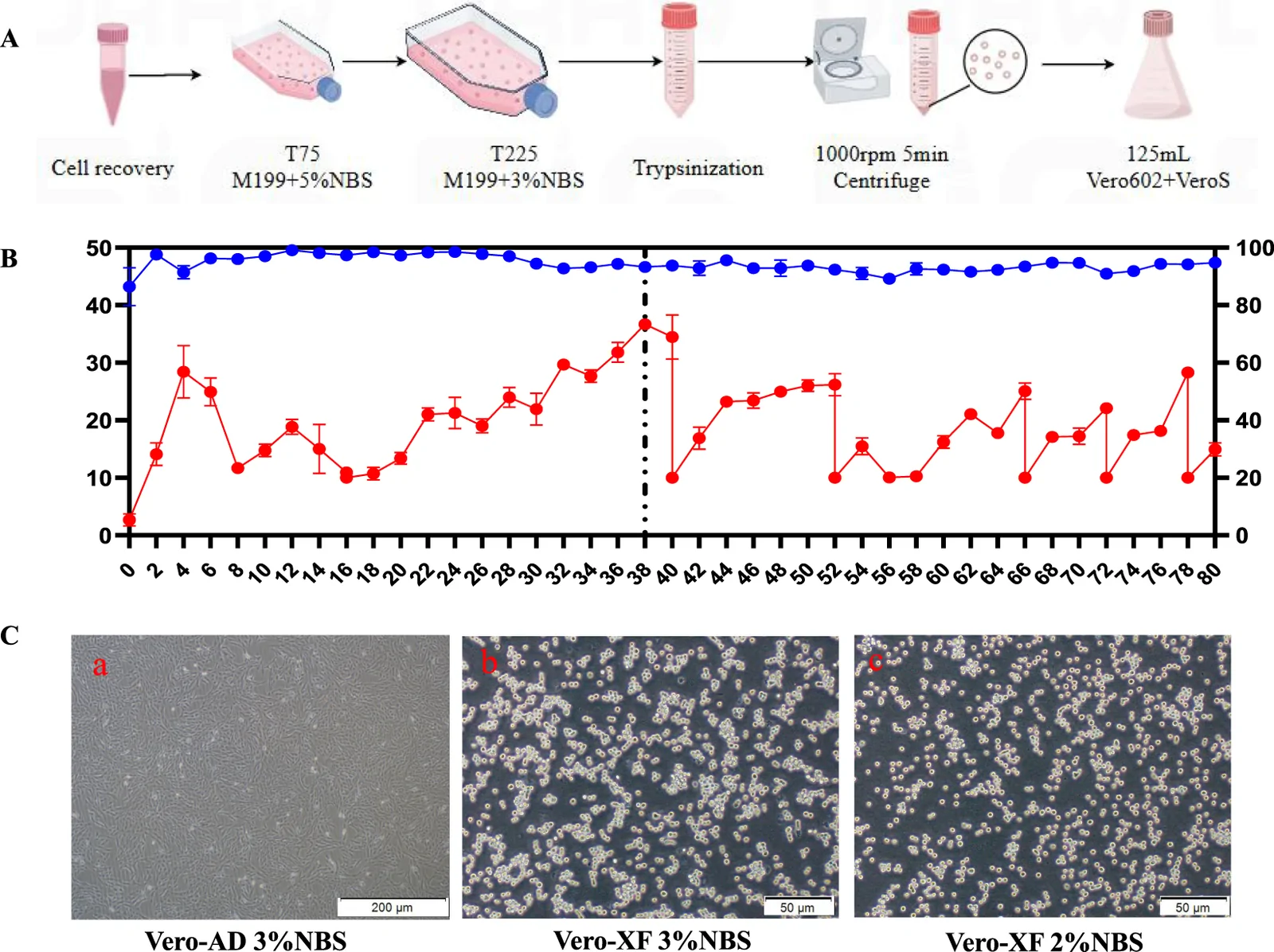
Establishment of a suspension Vero cell culture system. **(A)** Schematic illustration of the adaptation process from adherent to suspension Vero cells. **(B)** Growth curves of viable cell density and viability during suspension culture; red line represents viable cell density and blue line represents cell viability. **(C)** Morphological comparison of adherent and suspension-adapted Vero cells cultured under different serum concentrations, observed by optical microscopy.

The adaptation process (Figure 1A) and growth density curves showed that Vero cells experienced marked fluctuations in cell density and viability during the early stages of suspension adaptation. This response was likely attributable to simultaneous adaptation to both the suspension growth state and the new culture medium. Consequently, the initial adaptation phase was relatively prolonged. During the intermediate stage, cell density stabilized and viability remained consistently above 90%. After 38 days of suspension adaptation, cells achieved rapid proliferation within 4–6 days. Following six consecutive passages, cell density remained stable and viability consistently exceeded 90% (Figure 1B). Morphological observations further confirmed successful adaptation. Adherent Vero cells exhibited typical epithelial-like morphology, whereas suspension-adapted cells displayed a uniform suspension phenotype under 3% newborn bovine serum (NBS), 2% NBS, and post-thaw conditions, without residual adherent growth (Figure 1C). These findings demonstrate the successful conversion of adherent Vero cells into low-serum suspension cells.

### Biological characterization

**FIGURE 2:**
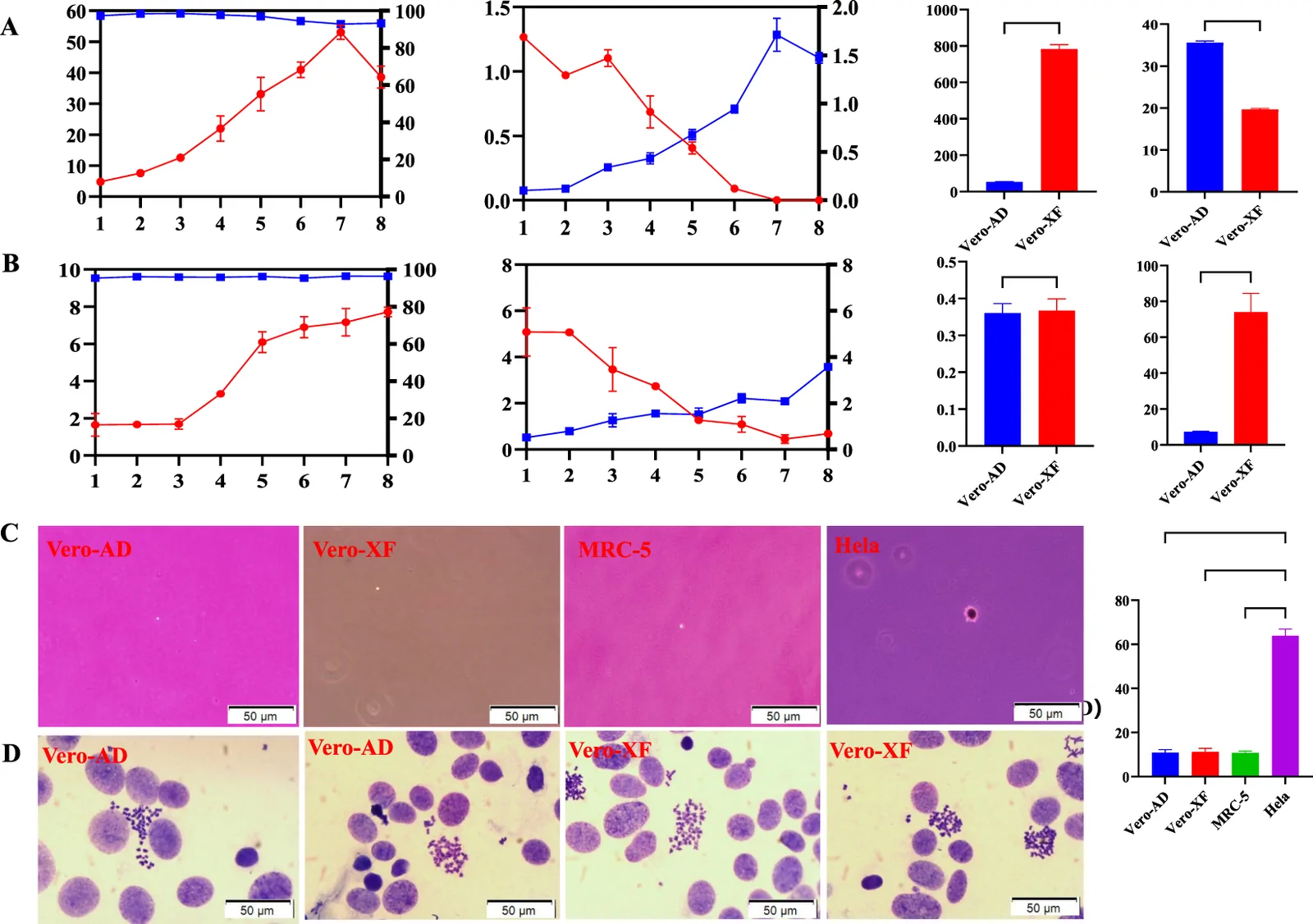
Biological characterization of adherent and suspension Vero cells. **(A)** Growth and metabolic profiles of adherent Vero cells, including cell density, cell viability, residual glucose concentration, and lactate accumulation. **(B)** Growth and metabolic profiles of suspension Vero cells. Red lines represent cell density and glucose concentration; blue lines represent cell viability and lactate concentration. This panel also includes histograms of growth kinetic parameters derived from growth curves (maximum cell density, population doubling time, and specific growth rate) and a quantitative comparison of viable cell density between groups on day 7. **(C)** Soft agar colony formation assay evaluating the *in vitro* clonogenic potential of adherent and suspension Vero cells, with MRC-5 cells as the negative control and HeLa cells as the positive control. Colonies containing >50 cells were considered effective. Colony formation rate (%) = average number of effective colonies/total number of seeded cells per well × 100%. Three replicates were analyzed per group. **(D)** Chromosome number distribution of adherent and suspension Vero cells. Data are presented as mean ± standard deviation (*n* = 3 biological replicates).; *p < 0.05, **p < 0.01, ***p < 0.001.

Comparison of the growth and metabolic characteristics of adherent and suspension Vero cells showed that suspension-adapted cells exhibited greatly enhanced proliferative capacity. Cell density increased more than 14-fold within 7 days, and viability remained consistently above 90% throughout the culture period. Metabolic analysis revealed complete glucose depletion in adherent cultures, whereas residual glucose remained in suspension cultures. Additionally, lactate accumulation was substantially higher in suspension cells, indicating enhanced glycolytic activity and distinct glucose utilization patterns compared with adherent cells (Figures 2A,B). Soft agar colony formation assays demonstrated that suspension Vero cells, similar to the negative controls (MRC-5 and Vero-AD cells), lacked tumorigenic characteristics, whereas positive control HeLa cells formed distinct colonies (Figure 2C). Chromosome analysis showed that suspension Vero cells maintained a stable chromosome number without abnormal chromosomal variations relative to adherent Vero cells. These findings indicate that the genetic characteristics of Vero cells remained stable throughout the adaptation process; there was no evidence of nuclear abnormalities (Figure 2D; Table 1).

**TABLE 1:** Chromosome analysis.

Cells	Chromosome number (X = 50)								
Vero-AD	<49	50	52	54	56	58	60	>61	2n = 50–60 (%)
	9	6	14	12	18	4	5	5	68
Vero-XF	<49	50	52	54	56	58	60	>61	2n = 50–60 (%)
	5	4	15	17	16	6	10	4	73

### Recombinant rabies VSV

**FIGURE 3:**
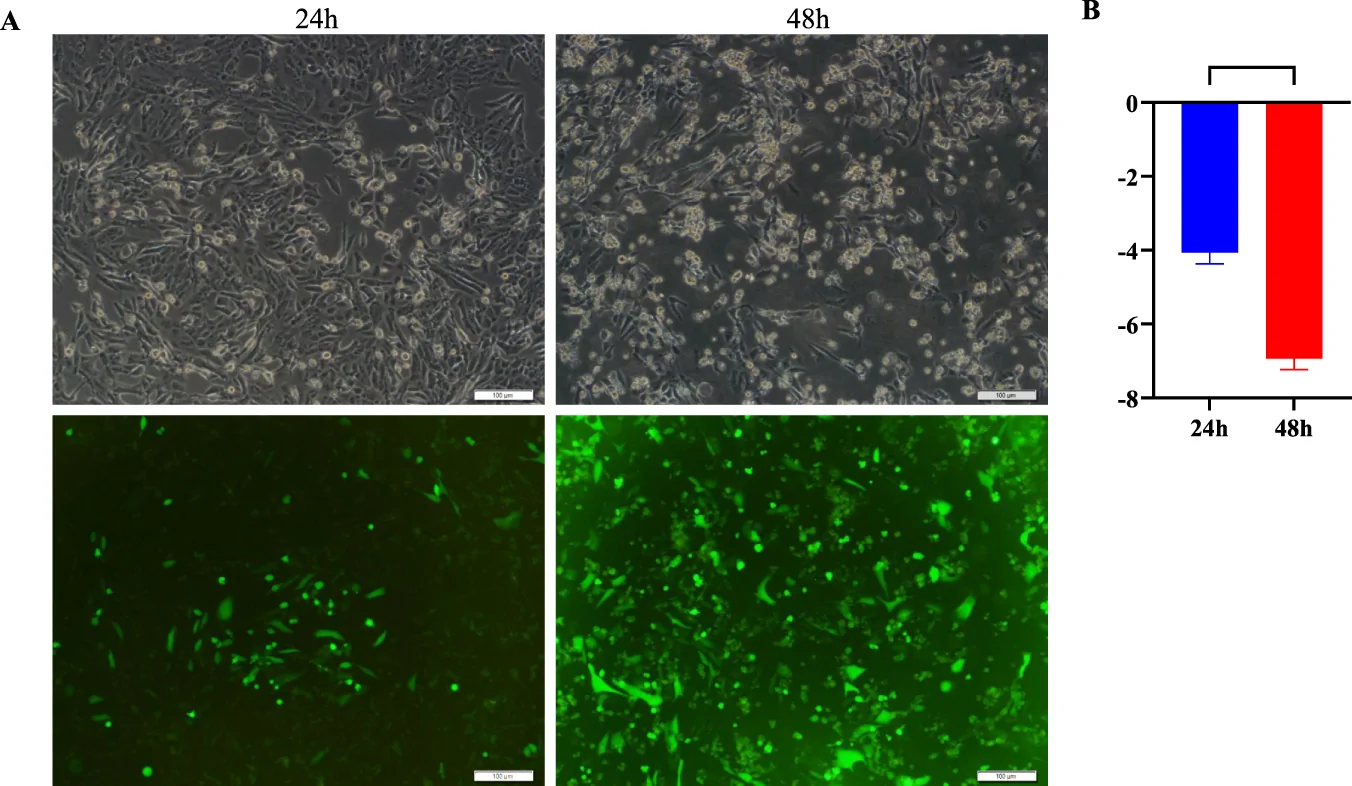
Titer analysis of recombinant rabies VSV in Vero cells. **(A)** Cytopathic morphology under bright-field microscopy (upper row) and GFP fluorescence imaging (lower row) of Vero cells at 24 h and 48 h after recombinant rabies VSV infection. **(B)** Progeny virus collected at various time points was quantified using the TCID_50_ assay. The y-axis represents log_10_ TCID_50_/0.1 mL, illustrating changes in viral replication titer over time. Data are presented as mean ± standard deviation (*n* = 3 biological replicates). *p < 0.05, **p < 0.01, ***p < 0.001.

Following infection of adherent Vero cells with a green fluorescent protein (GFP)-labeled recombinant VSV, only mild cytopathic effects were observed at 24 h; GFP-positive cells sporadically appeared (Figure 3A). The progeny virus titer at this time point was TCID_50_ = 10^−4^/0.1 mL. By 48 h, extensive cytopathic effects had developed, accompanied by pronounced increases in GFP-positive cell number and fluorescence intensity. Viral titers increased to TCID_50_ = 10^−7^/0.1 mL (Figure 3B). These results demonstrate that the recombinant VSV efficiently replicates and proliferates in adherent Vero cells during infection.

### Metabolomic analysis and pathway enrichment

**FIGURE 4:**
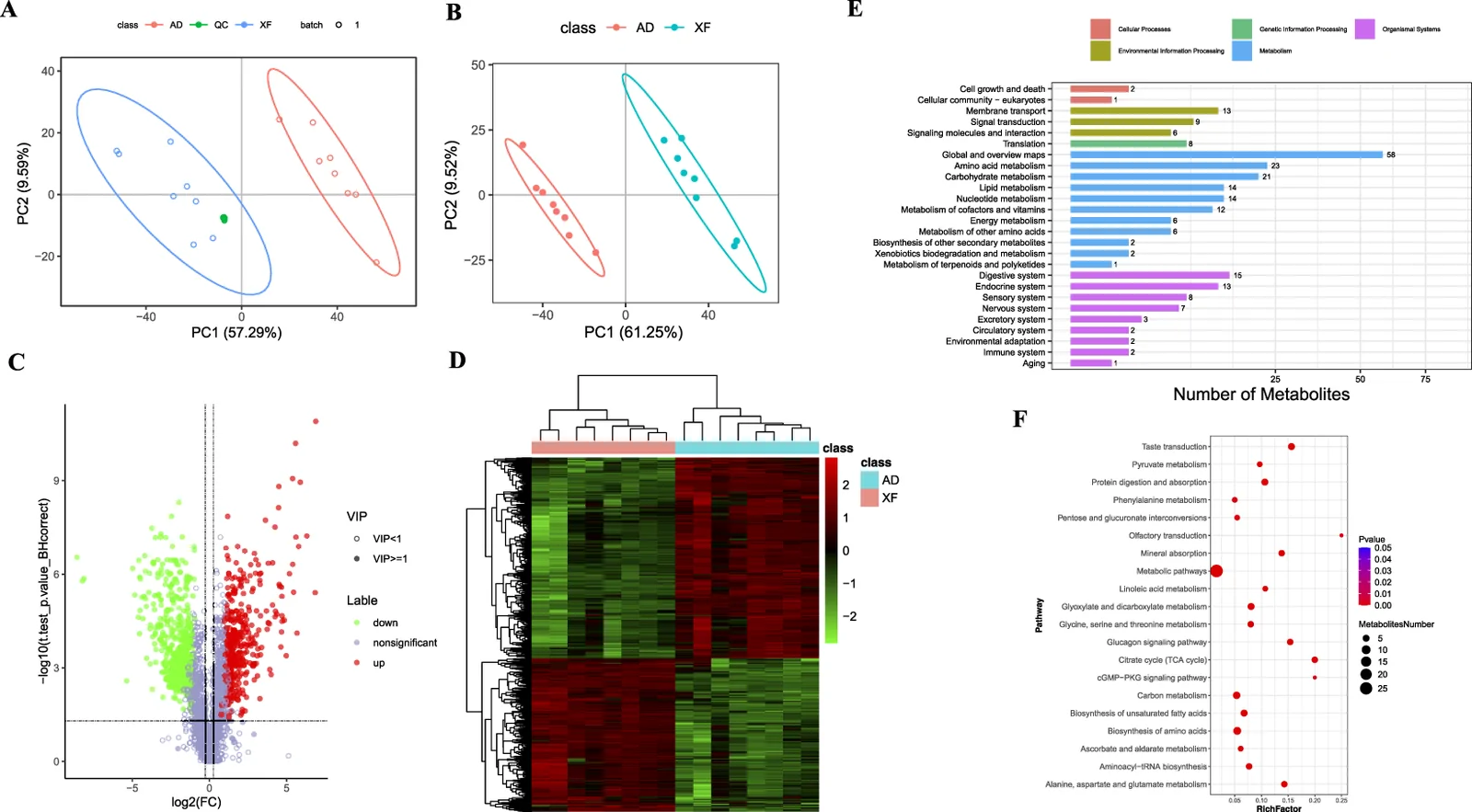
Metabolomic analysis and pathway enrichment under negative-ion (neg) mode. **(A)** Principal component analysis (PCA) score plot of all samples in neg mode; ellipses indicate the 95% confidence interval for each group. **(B)** PCA score plot of the AD and XF groups after removal of quality control (QC) samples. **(C)** Volcano plot of differential metabolites between the AD and XF groups. Red dots indicate significantly upregulated metabolites, green dots indicate significantly downregulated metabolites, and gray dots indicate metabolites without significant differences (screening criteria: VIP ≥1 and adjusted p < 0.05). **(D)** Hierarchical clustering heatmap of differential metabolites showing metabolite expression patterns across samples; colors represent normalized abundance (red, high expression; green, low expression). **(E)** Kyoto Encyclopedia of Genes and Genomes (KEGG) functional classification of identified metabolites. The y-axis represents functional categories, and the x-axis represents the number of metabolites within each category. **(F)** KEGG pathway enrichment analysis of differential metabolites. The x-axis represents the enrichment factor, and the y-axis represents enriched pathways. Dot color corresponds to p value, and dot size indicates the number of enriched differential metabolites. Data are presented as mean ± standard deviation (*n* = 3 biological replicates). *p < 0.05, **p < 0.01, ***p < 0.001.

Metabolomic analysis in neg mode showed that PCA and PLS-DA produced tight clustering within groups and clear separation between adherent and suspension Vero cells, indicating substantial differences in metabolic profiles between the two culture conditions (Figures 4A,B). Volcano plots and hierarchical clustering also demonstrated distinct metabolic signatures: 343 upregulated and 448 downregulated metabolites were identified in suspension cells relative to adherent cells (Figures 4C,D). KEGG pathway enrichment analysis revealed that differential metabolites were primarily associated with carbon metabolism, amino acid metabolism (including alanine/aspartate/glutamate metabolism and glycine/serine/threonine metabolism), lipid metabolism, and the TCA cycle (Figures 4E,F). These findings suggest that Vero cells undergo extensive reprogramming of carbon/energy, amino acid, and lipid metabolism during suspension adaptation, providing metabolic support for stable growth and rapid proliferation under suspension culture conditions.

**FIGURE 5:**
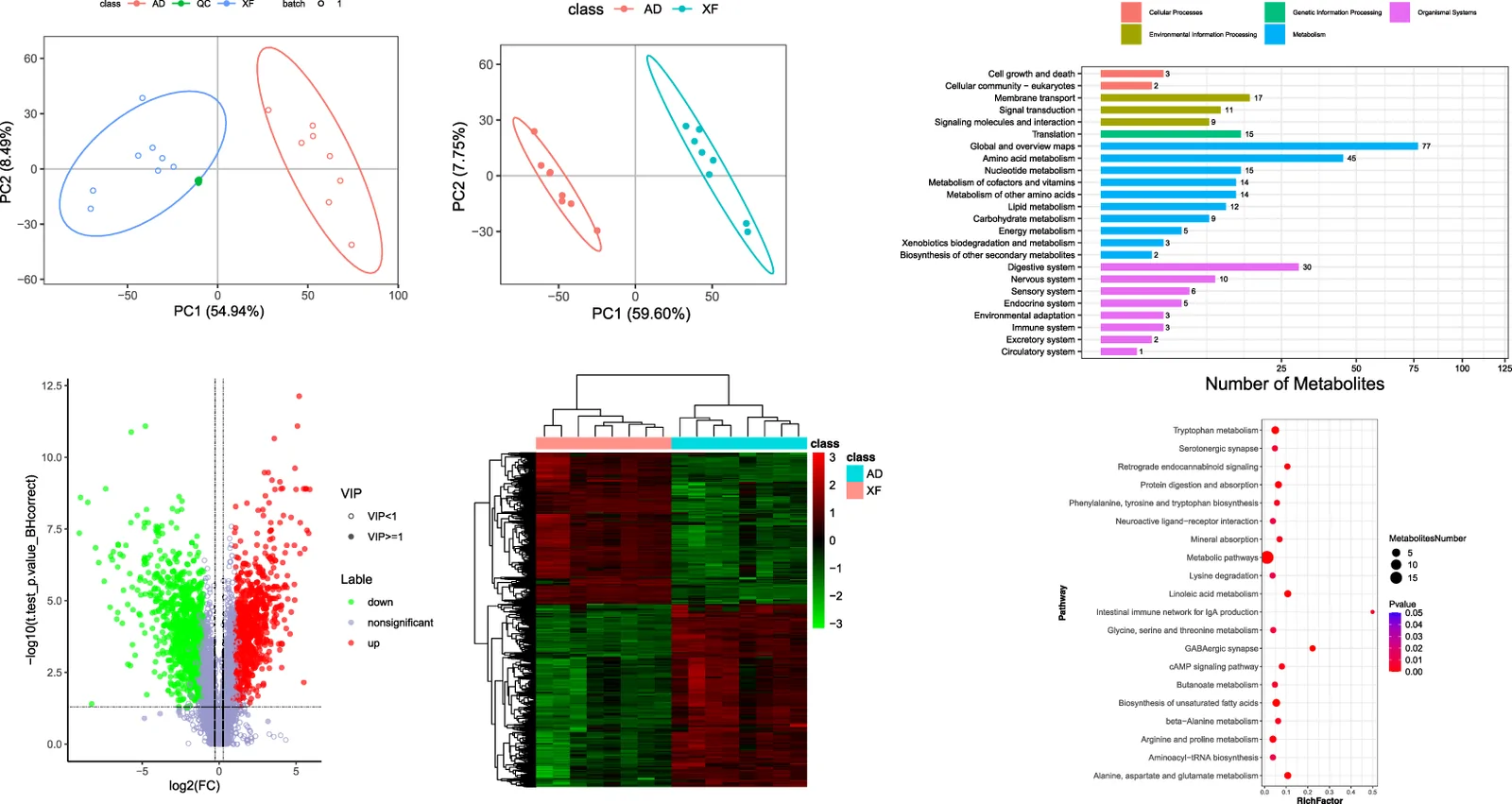
Metabolomic analysis and pathway enrichment under positive-ion (pos) mode. **(A)** Principal component analysis (PCA) score plot of all samples in pos mode; ellipses represent the 95% confidence interval for each group. **(B)** PCA score plot of the AD and XF groups after removal of quality control (QC) samples. **(C)** Volcano plot of differential metabolites between the AD and XF groups. Red dots indicate significantly upregulated metabolites, green dots indicate significantly downregulated metabolites, and gray dots indicate metabolites without significant differences (screening criteria: VIP ≥1 and adjusted *p* < 0.05). **(D)** Hierarchical clustering heatmap of differential metabolites showing metabolite expression patterns across samples; colors represent normalized metabolite abundance (red, high expression; green, low expression). **(E)** Kyoto Encyclopedia of Genes and Genomes (KEGG) functional classification of identified metabolites. The y-axis represents functional categories, and the x-axis represents the number of metabolites within each category. **(F)** KEGG pathway enrichment analysis of differential metabolites. The x-axis represents the enrichment factor, and the y-axis represents enriched pathways. Dot color corresponds to p value, and dot size indicates the number of enriched differential metabolites. Data are presented as mean ± standard deviation (*n* = 3 biological replicates). *p < 0.05, **p < 0.01, ***p < 0.001.

In pos mode, both PCA and PLS-DA showed tight clustering within groups and clear separation between adherent and suspension Vero cells, confirming substantial metabolic differences between the two culture conditions (Figures 5A,B). Volcano plot and clustering analyses identified 777 upregulated and 940 downregulated metabolites in suspension cells relative to adherent cells, with highly consistent expression patterns across samples (Figures 5C,D). KEGG pathway enrichment analysis indicated that differential metabolites were primarily associated with amino acid metabolism, including tryptophan metabolism and phenylalanine/tyrosine/tryptophan biosynthesis, as well as lipid metabolism and neuroactive ligand–receptor interaction pathways, complementing the findings from neg mode analysis (Figures 5E,F).

### Integrated metabolomic analysis

To maximize coverage of metabolites with diverse ionization properties and minimize mode-specific detection bias, data from both pos and neg modes were integrated for untargeted metabolomic analysis. This combined approach provided a more comprehensive characterization of the systemic metabolic reprogramming associated with suspension adaptation in Vero cells.

**FIGURE 6:**
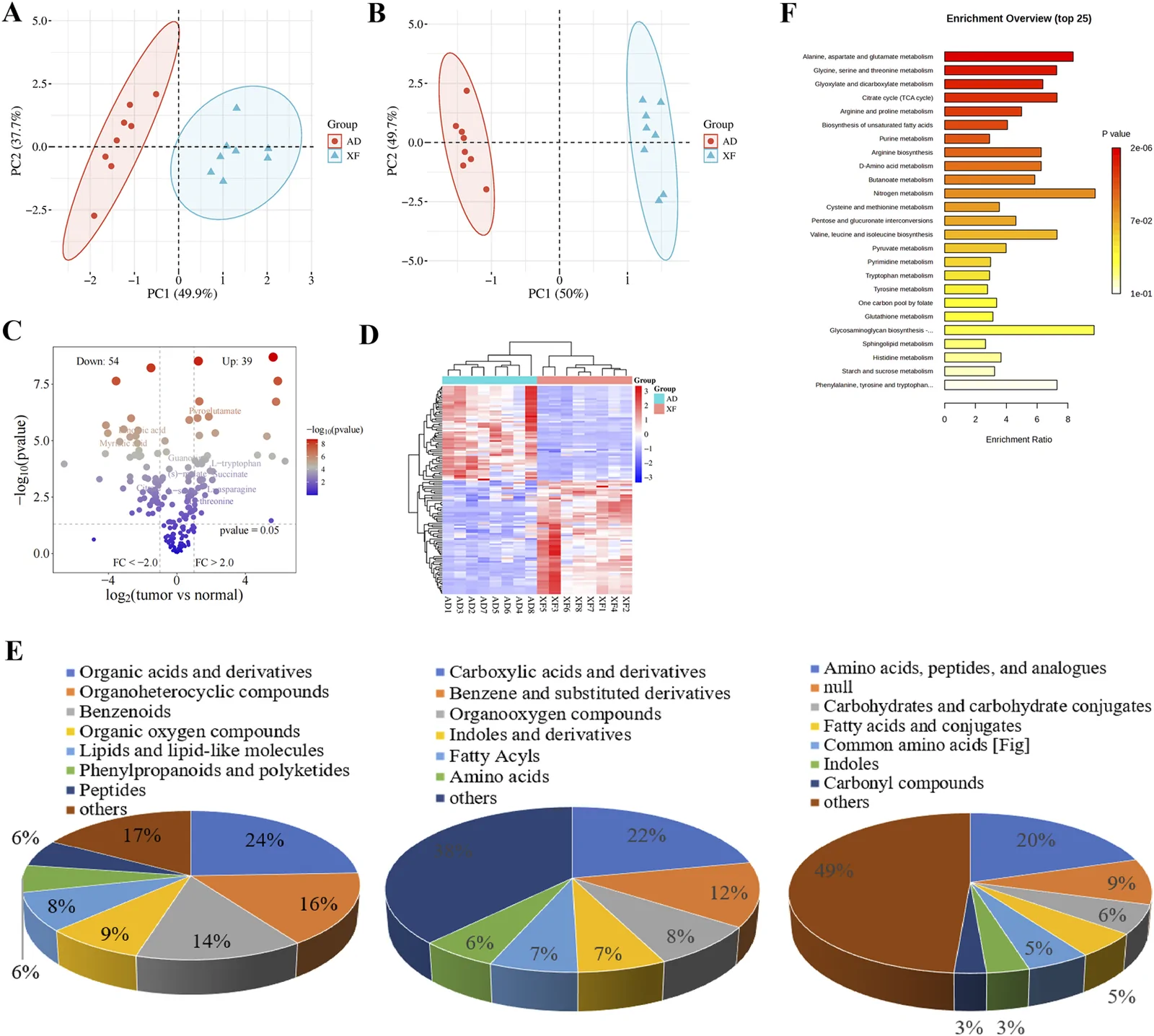
Integrated metabolomic analysis. **(A)** Principal component analysis (PCA) score plot of all samples; ellipses indicate the 95% confidence interval for each group. **(B)** Partial least squares-discriminant analysis (PLS-DA) of all samples. **(C)** Volcano plot of differential metabolites. Red dots indicate significantly upregulated metabolites, blue dots indicate significantly downregulated metabolites, and gray dots indicate metabolites without significant differences. **(D)** Hierarchical clustering heatmap of differential metabolites showing expression patterns across samples. The color scale represents normalized metabolite abundance (red, high abundance; blue, low abundance). **(E)** Human Metabolome Database (HMDB)(Wishart et al., 2018) hierarchical classification of differential metabolites. **(F)** Kyoto Encyclopedia of Genes and Genomes (KEGG) pathway enrichment analysis of differential metabolites. Bubble size represents the enrichment factor, and color intensity corresponds to the *p*-value; darker red indicates a smaller *p*-value. Data are presented as mean ± standard deviation (*n* = 3 biological replicates). *p < 0.05, **p < 0.01, ***p < 0.001.

PCA of the integrated metabolomic dataset showed clear separation between adherent and suspension culture groups, whereas QC samples clustered tightly, indicating substantial metabolic differences between the two culture modes (Figure 6A). PLS-DA further improved group discrimination and identified metabolite variables that most strongly contributed to these differences, helping define major sources of metabolic variation (Figure 6B). Using the criteria of fold change ≥2, *p* < 0.05, and VIP ≥1, 119 differential metabolites were identified, including 32 upregulated and 47 downregulated metabolites (Figure 6C). Hierarchical clustering demonstrated highly consistent expression patterns among biological replicates within each group and clear separation between adherent and suspension cultures. Differential metabolites formed two major expression clusters, reflecting the distinct biological states of the two culture conditions. Based on the Human Metabolome Database hierarchical classification system, differential metabolites were annotated at the Super Class (Level 2), Class (Level 3), and Subclass (Level 4) levels. At the Super Class level, organic acids and derivatives comprised 24% of differential metabolites, followed by organic heterocyclic compounds (16%) and benzenoids (14%). At the Class level, carboxylic acids and derivatives represented 22%, followed by substituted benzene derivatives (12%) and organic oxygen compounds (8%). At the Subclass level, amino acids, peptides, and their analogs constituted 20%; carbohydrates and their conjugates, fatty acid conjugates, and other subclasses were more evenly distributed (Figure 6E). Overall, differential metabolites associated with the transition from adherent to suspension growth were predominantly organic acids and amino acid-related compounds, highlighting extensive metabolic reprogramming centered on organic acid and amino acid metabolism during cellular adaptation to suspension culture.

Pathway enrichment analysis of differential metabolites between adherent and suspension Vero cells revealed that the most strongly enriched pathways included alanine, aspartate, and glutamate metabolism; glycine, serine, and threonine metabolism; glyoxylate and dicarboxylate metabolism; and the TCA cycle, all of which exhibited extremely low *p*-values (Figure 6F). Additional significantly enriched pathways included arginine and proline metabolism, unsaturated fatty acid biosynthesis, and purine metabolism. The findings indicate that suspension adaptation in Vero cells involves extensive metabolic reprogramming centered on amino acid and carbon metabolism, accompanied by coordinated changes in lipid and nucleotide metabolism. Collectively, the results reflect adaptive metabolic remodeling of energy production, biosynthetic processes, and oxidative stress responses that support growth under suspension culture conditions.

### Differential metabolite landscapes

Functional annotation and comprehensive pathway enrichment analysis were performed on the differential metabolites. By integrating pathway significance and enrichment factors, 23 key differential metabolites were identified (Table 2). These metabolites are involved in major pathways related to energy, amino acid, nucleotide, and lipid metabolism, highlighting the core metabolic alterations associated with Vero cell adaptation to suspension culture.

**TABLE 2:** Differentially abundant metabolites.

Compound ID	Metabolite names	Mode	log_2_FC	*p*-value	VIP	Level
0.643_134.0215	(s)-Malate	neg	1.345135337	0.0006	1.0315	level1
1.892_283.0912	Guanosine	neg	1.206681014	0.0006	1.0561	level1
0.66_105.0426	L-serine	neg	1.491537112	0.0002	1.1123	level1
0.661_132.0535	L-asparagine	neg	1.595040896	0.0002	1.1323	level1
0.954_323.0519	Cytidine5′-monophosphate	pos	1.445130212	0.0002	1.1446	level1
0.759_119.0583	L-threonine	neg	1.641627207	0.0003	1.1542	level1
0.644_192.0268	Citrate	neg	−1.269895409	0.0011	1.2014	level1
0.659_118.0266	Succinate	neg	2.076588022	0.0002	1.3161	level1
2.977_204.0896	L-tryptophan	neg	1.931516275	0.0002	1.3219	level1
1.136_129.0426	Pyroglutamate	pos	1.858596811	0.0002	1.3549	level1
0.954_347.063	Deoxyguanosine monophosphate	pos	−2.14557117	0.0011	1.364	level1
3.037_219.1105	Pantothenic acid	pos	1.779714333	0.0003	1.424	level1
0.7_131.0695	Creatine	pos	−2.745472224	0.0002	1.6247	level1
0.647_141.0191	Ethanolamine phosphate	neg	5.891429391	0.0002	2.4083	level1
0.635_88.016	Pyruvic acid	neg	1.345135337	0.0006	1.0315	level2
0.627_230.0186	D-ribulose 5-phosphate	neg	−1.695987675	0.0002	1.2263	level2
0.638_185.9928	3-Phosphoglyceric acid	neg	−2.201729282	0.0148	1.3295	level2
0.994_347.0626	Adenosine 5′-monophosphate	neg	−1.909443963	0.0047	1.3466	level2
9.606_280.2395	Linoleic acid	neg	−2.40228942	0.0002	1.5314	level2
9.358_228.2084	Myristic acid	neg	−2.740599168	0.0002	1.6299	level2
8.891_278.2245	Gamma-linolenic acid	pos	−2.680487931	0.0002	1.6496	level2
9.573_304.2395	Arachidonic acid	neg	−3.342442133	0.0002	1.8449	level2
9.524_328.2396	Docosahexaenoic acid	neg	−4.184443565	0.0002	2.0657	level2

Level 1 indicates metabolite identification confirmed with authentic standards. Level 2 indicates putative identification based on MS/MS, spectra and database matching.

#### Energy metabolism

**FIGURE 7:**
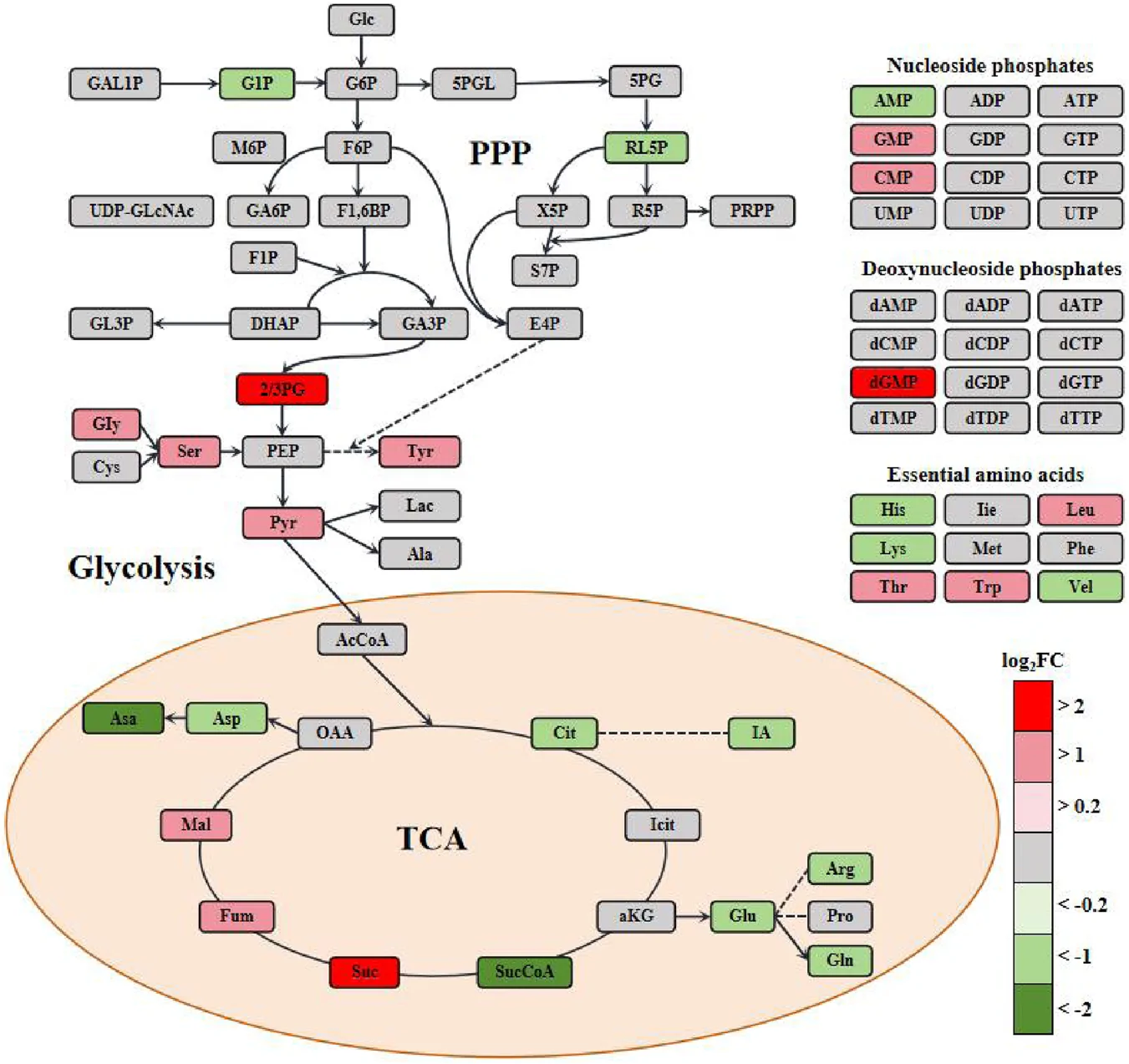
Differential analysis of energy metabolism and central carbon metabolism pathways in suspension and adherent Vero cells. Metabolites shown in red are significantly upregulated, whereas those shown in blue are significantly downregulated. Data are presented as mean ± standard deviation (*n* = 3 biological replicates).

Energy metabolism is fundamental to cellular function. It primarily encompasses glycolysis, the TCA cycle, and creatine phosphate metabolism, which provide ATP and reducing equivalents for processes such as proliferation, adhesion, and viral infection. Analysis of energy-related pathways identified pyruvate (Pyr), succinate (Suc), malate (Mal), citrate (Cit), creatine (Cr), 3-phosphoglycerate (3-PGA), and D-ribulose-5-phosphate (Ru5P) as key metabolites associated with cellular adaptation. Within the glycolytic pathway, levels of 3-phosphoglycerate and pyruvate were significantly increased. In the TCA cycle, succinate and malate levels were substantially elevated, whereas citrate levels were reduced. D-ribulose-5-phosphate, a key intermediate of the pentose phosphate pathway, was significantly decreased; creatine, an important energy-storage molecule, was also greatly reduced (Figure 7). These findings indicate enhanced glycolytic activity (Figures 2A–B) in suspension Vero cells, accompanied by accumulation of TCA cycle intermediates, reduced metabolites associated with mitochondrial oxidative metabolism, and decreased pentose phosphate pathway flux, collectively reflecting metabolic reprogramming. Notably, the accumulation of succinate and malate does not necessarily indicate impaired TCA cycle function but rather represents an adaptive metabolic response. Previous studies have shown that succinate inhibits prolyl hydroxylase activity, stabilizes the hypoxia-inducible factor (HIF)-1α pathway, suppresses apoptosis-related genes, and promotes proliferation-associated signaling, thereby enhancing resistance to apoptosis and environmental stress under suspension culture conditions (Atallah et al., 2022; Xu et al., 2025). Concurrently, lactate production mediated by lactate dehydrogenase and activation of the malate-aspartate shuttle promote cytosolic NAD^+^ regeneration and maintain the cytoplasmic NAD^+^/NADH redox balance. These processes collectively sustain glycolytic flux and provide a stable supply of reducing equivalents and energy to support rapid cell proliferation (Borst, 2020; Broeks et al., 2023).

#### Amino acid and nitrogen metabolism

Amino acid and nitrogen metabolism constitute core pathways for protein synthesis, signal transduction, oxidative stress regulation, and the production of biomolecular precursors for nucleotide and lipid synthesis. In this study, key metabolites identified were L-serine (L-Ser), L-asparagine (L-Asn), L-threonine (L-Thr), L-tryptophan (L-Trp), and pyroglutamate (Pyr-Glu). Among these, L-Ser was significantly upregulated; L-Asn, L-Thr, L-Trp, and Pyr-Glu were significantly downregulated. Under suspension culture conditions, Vero cells are exposed to fluid shear stress, lose extracellular matrix-dependent adhesion, and experience increased oxidative stress. To adapt to these microenvironmental changes, cells enhance antioxidant defenses and biosynthetic capacity through nitrogen metabolism reprogramming. As a key precursor for one-carbon metabolism, glutathione synthesis, and phospholipid biosynthesis, increased L-Ser levels promote redox homeostasis, support membrane and nucleotide synthesis, and improve cell survival and proliferation under stress conditions (Locasale, 2013). In contrast, the reduced levels of L-Asn, L-Thr, and L-Trp suggest decreased reliance on exogenous amino acids and a shift toward endogenous biosynthesis supported by glycolysis-derived carbon skeletons. This adaptation may optimize nitrogen utilization and metabolic efficiency during high-density suspension culture. Elevated L-Ser levels appear to be a central metabolic feature supporting high viability and rapid proliferation in suspension-adapted cells. Additionally, reduced flux through glutamine-anaplerotic pathways may redirect carbon toward glycolytic and biosynthetic processes, providing critical metabolic support for enhanced growth density and culture stability (Quesney et al., 2003).

#### Nucleotide metabolism

Nucleotide metabolism is central to DNA/RNA synthesis, energy metabolism, and signal transduction. Key differential metabolites identified in this study included guanosine (Guo), cytidine 5′-monophosphate (CMP), deoxyguanosine monophosphate (dGMP), and adenosine 5′-monophosphate (AMP). Changes in their abundance reflect adaptive adjustments in nucleotide biosynthesis and cellular energy regulation. As a precursor of purine nucleotides, Guo participates in DNA/RNA synthesis, guanosine triphosphate–dependent energy metabolism, and signal transduction. Its robust upregulation suggests an increased demand for purine nucleotide production to support cell proliferation. CMP, a key intermediate in pyrimidine metabolism, provides precursors for both RNA synthesis and membrane phospholipid biosynthesis. Elevated CMP levels indicate enhanced pyrimidine metabolism in suspension cells, supporting rapid growth and proliferation (Kent et al., 1991). AMP, a central regulator of cellular energy status and the AMPK signaling pathway, was significantly decreased, suggesting an increased ATP/AMP ratio and a more energy-abundant intracellular environment. This finding is consistent with the enhanced glycolytic activity observed in suspension-adapted cells. In contrast, dGMP, an essential precursor for DNA synthesis, was significantly reduced, indicating either decreased DNA synthesis activity or altered deoxyribonucleotide flux. Overall, nucleotide metabolism in suspension Vero cells exhibited a reprogrammed pattern characterized by increased pyrimidine nucleotide precursors and decreased purine deoxyribonucleotides and energy-sensing nucleotides. This metabolic configuration may prioritize RNA biosynthesis and energy homeostasis while moderating DNA synthesis, thereby supporting proliferation and survival under suspension culture conditions (Jang et al., 2022).

#### Lipid metabolism

**FIGURE 8:**
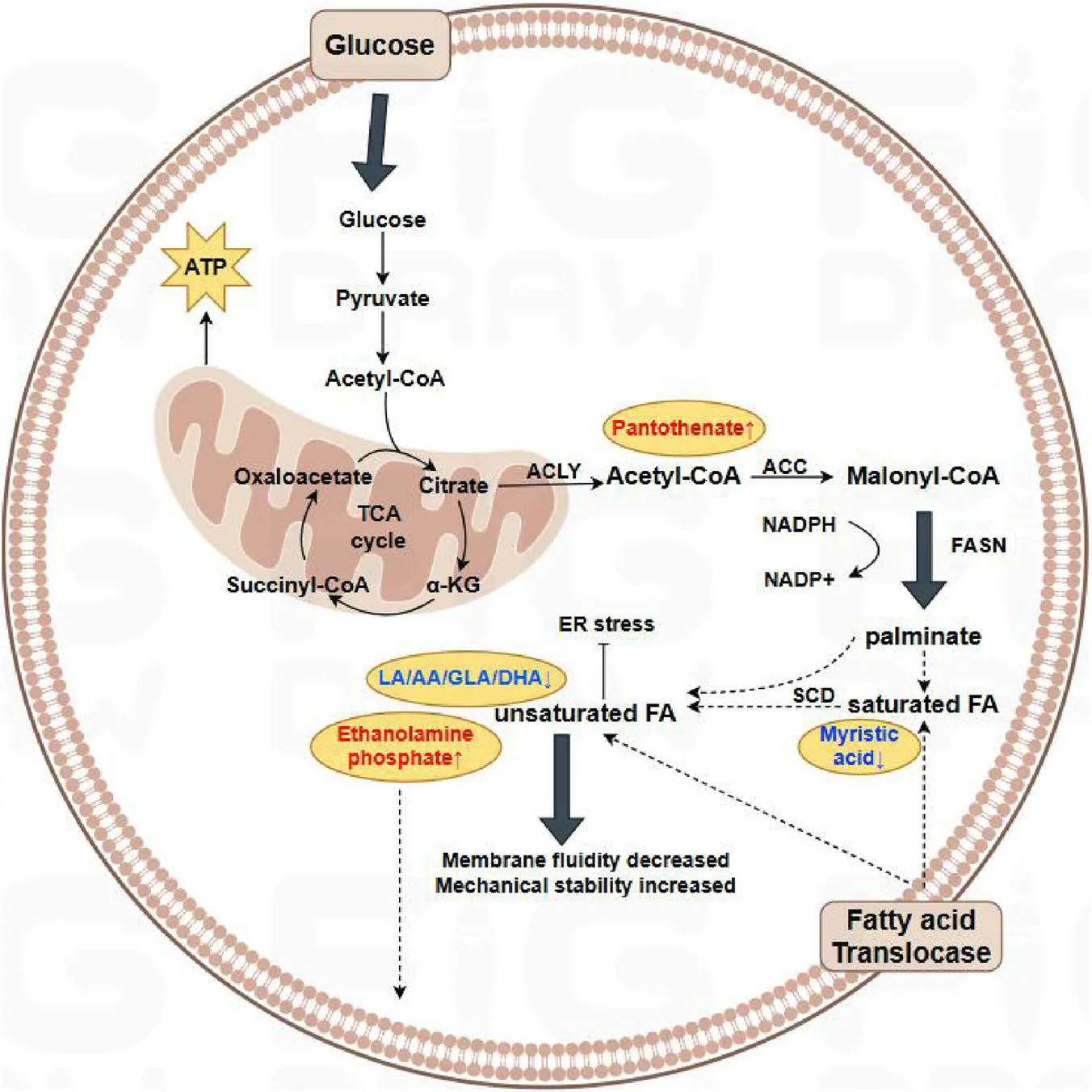
Schematic illustration of fatty acid synthesis and cell membrane remodeling pathways in Vero cells. Metabolites shown in red are significantly upregulated, whereas those shown in blue are significantly downregulated. Data are presented as mean ± standard deviation (*n* = 3 biological replicates).

Lipid metabolism is central to cell membrane synthesis, energy storage, and signal transduction, and also contributes to cell adhesion, immune regulation, and viral infection. Key differential metabolites identified in this pathway were linoleic acid (LA), γ-linolenic acid (GLA), arachidonic acid (AA), docosahexaenoic acid (DHA), myristic acid (MA), ethanolamine phosphate (EAP), and pantothenic acid (PA). Changes in their abundance reflect adaptive remodeling of membrane structure, inflammatory signaling, and energy metabolism. In suspension cells, polyunsaturated fatty acids (PUFAs), including LA, GLA, AA, and DHA, were significantly downregulated, whereas EAP, a precursor of phospholipid biosynthesis, was substantially upregulated. From a membrane mechanics perspective, high PUFA content increases membrane fluidity while reducing mechanical stability. Conversely, decreasing PUFA content and increasing the relative proportion of saturated fatty acids can reduce membrane fluidity, enhance membrane rigidity, and improve resistance to shear stress, thus reducing membrane damage and apoptosis during suspension culture. This mechanism represents a key membrane remodeling strategy that facilitates adaptation to the suspension environment (Butler et al., 2001; Kadri et al., 2021) (Figure 8). Additionally, PUFAs serve as precursors of inflammatory mediators such as prostaglandins. Their pronounced downregulation can suppress inflammatory signaling pathways (Calder, 2010) and reduce cellular immune activation, potentially creating conditions favorable for viral infection (Yang et al., 2026). Among saturated fatty acids, MA was significantly reduced. Given that MA is required for protein myristoylation, a modification involved in membrane localization and signal transduction, its reduction may alter the function of adhesion-related proteins and contribute to the reduced adhesion phenotype of suspension cells (Kunisawa et al., 2007). EAP, a key precursor for phospholipid synthesis, was strongly upregulated, potentially supporting increased membrane biogenesis during cell proliferation and facilitating membrane remodeling required for suspension growth (van Meer et al., 2008). Pantothenic acid, a precursor of coenzyme A, was also elevated, providing coenzyme support for lipid biosynthesis, the TCA cycle, and other metabolic pathways involved in suspension-associated metabolic reprogramming.

## Discussion

Using untargeted metabolomics, we systematically characterized the intracellular metabolic profiles of Vero cells under adherent, suspension, and integrated culture conditions. Through multivariate statistical analysis, differential metabolite screening, and KEGG pathway enrichment analysis, this study comprehensively revealed the metabolic reprogramming mechanisms that enable Vero cells to adapt to suspension culture. These findings provide a theoretical foundation and valuable data for the optimization and industrial application of Vero cell suspension culture systems in viral vaccine production.

At the level of energy metabolism, suspension-adapted Vero cells exhibited Warburg-like metabolic characteristics, including enhanced glycolysis, increased lactate accumulation, and altered distributions of TCA cycle intermediates (Vaupel and Multhoff, 2021). Similar metabolic features have been reported during the suspension adaptation of other industrially relevant mammalian cell lines, including HEK293 (Jang et al., 2022) and CHO cells (Morrissey et al., 2026). This pattern appears to represent a conserved adaptive strategy that enables cells to transition from substrate-dependent growth to agitated suspension culture while rapidly providing energy and biosynthetic precursors required for proliferation. Moreover, this study identified synergistic alterations in key metabolic intermediates (e.g., pyruvate, 3-phosphoglycerate, and D-ribulose-5-phosphate), revealing substantial flux redistribution across glycolysis, the TCA cycle, and the pentose phosphate pathway. These findings provide a comprehensive framework for the targeted regulation and optimization of central carbon metabolism. At the level of amino acid metabolism, significant upregulation of L-Ser constituted a hallmark of Vero cell adaptation to suspension culture. By participating in one-carbon metabolism, glutathione synthesis, and phospholipid biosynthesis, serine enhances antioxidant capacity and biosynthetic efficiency, representing a common metabolic response to loss of adhesion, increased oxidative stress, and the demands of rapid proliferation (Locasale, 2013; Ducker and Rabinowitz, 2017; Pan et al., 2021). To our knowledge, this work is the first to report the simultaneous downregulation of L-Asn, L-Thr, and L-Trp in Vero cells. This result suggests reduced reliance on exogenous amino acids and a shift toward endogenous biosynthesis, thereby improving nitrogen utilization efficiency. These results may provide new directions for optimizing amino acid composition in serum-free media (Salazar et al., 2016; Rong et al., 2023). At the level of nucleotide metabolism, suspension Vero cells exhibited increased levels of guanosine and cytidine 5′-monophosphate, accompanied by decreased levels of deoxyguanosine monophosphate and AMP. This pattern reflects a metabolic strategy that prioritizes RNA synthesis and energy homeostasis. Given the well-established capacity of Vero cells to support viral propagation, this nucleotide allocation strategy may not only satisfy cellular proliferative demands but also provide substrates for viral replication, offering new insights into the coordinated metabolic requirements of high-yield cell and virus production systems (Bhutta et al., 2021). Regarding lipid and membrane metabolism, PUFAs were significantly reduced in suspension-adapted Vero-XF cells compared with adherent Vero-AD cells, whereas phosphoethanolamine levels were substantially elevated. This metabolic shift is expected to decrease membrane fluidity, increase membrane mechanical stability, and improve cellular tolerance to shear stress (Kadri et al., 2021; Dawczynski et al., 2022). To our knowledge, this metabolic profile has not been identified in previous metabolomic studies of suspension-adapted Vero cells or related cell types. This mechanism not only explains the successful adaptation of Vero cells to carrier-free suspension culture but also identifies potential metabolic targets for optimizing medium lipid composition and improving shear stress tolerance. Notably, our untargeted metabolomic data revealed that suspension-adapted Vero cells exhibit metabolic signatures frequently associated with malignant cells, including Warburg-like aerobic glycolysis, succinate accumulation, and extensive membrane-lipid remodeling. Nevertheless, soft-agar colony-formation assays demonstrated that these suspension-adapted Vero cells remain non-tumorigenic and lack anchorage-independent clonogenic capacity. Together, these observations imply that such metabolic reprogramming is not a unique hallmark of oncogenic transformation. Instead, these metabolic phenotypes likely represent adaptive physiological responses of Vero cells to extrinsic cell-culture stressors, such as high cell density, hydrodynamic shear force, and nutrient limitation under suspension cultivation conditions (Vazquez and Oltvai, 2011; Liberti and Locasale, 2016; Abdel-Haleem et al., 2017).

This study has three prominent limitations. First, a single omics technique was used: only untargeted LC-MS/MS metabolomics was applied to analyze intracellular small-molecule metabolic alterations, without integrated transcriptomic and proteomic data. Therefore, the upstream gene and protein regulatory events driving changes in metabolic pathways cannot be explained, and only phenotypic metabolic differences can be described. Second, the culture system was limited to a small scale. Suspension adaptation, growth assays, and metabolomic sample collection were all performed in 125-mL shake flasks. The metabolic signatures identified here were not validated in high-density bioreactor cultures—shake flasks substantially differ from industrial-scale fermentation systems with respect to shear stress, dissolved oxygen, nutrient consumption, and pH control. Complementing our glucose and lactate observations (Figures 2A,B), the shake-flask system lacked closed-loop pH regulation. Under non-pH-controlled conditions, mammalian cell cultures may undergo a metabolic switch to lactate co-consumption that supports cell proliferation (Martínez-Monge et al., 2019). Therefore, we cannot determine whether a similar lactate reutilization process occurs in suspension-adapted Vero cells under our experimental conditions. Further studies using pH-controlled stirred bioreactors are needed to clarify this metabolic feature. Third, functional verification experiments were not conducted. Although differential metabolites were screened and enriched metabolic pathways were identified, this study did not use strategies such as exogenous supplementation of key metabolites or gene editing of critical metabolic enzymes to directly confirm the regulatory effects of L-serine, ethanolamine phosphate and polyunsaturated fatty acids on cellular shear resistance and suspension proliferation capacity.

In summary, this study validated conserved features of Vero cell adaptation to suspension culture, such as Warburg-like metabolism, L-serine upregulation, and directed nucleotide allocation; it also identified Vero cell-specific adaptations, including nitrogen metabolism redistribution and coordinated membrane lipid remodeling. Based on these metabolic characteristics, we propose a multidimensional optimization strategy: (1) at the medium level, increase L-Ser and ethanolamine phosphate availability while reducing PUFA content; (2) at the supplementation level, control glucose concentrations, implement phased glutamine supplementation, and add selected organic acids to alleviate lactate-associated toxicity; and (3) at the genetic level, overexpress serine biosynthesis and phospholipid synthesis enzymes while suppressing fatty acid desaturase and lactate dehydrogenase expression. These approaches provide clear targets for developing shear-resistant, highly stable cell lines.

## Data Availability

The original contributions presented in the study are included in the article/supplementary material, further inquiries can be directed to the corresponding author.
